# Quality and Shelf-Life Modeling of Frozen Fish at Constant and Variable Temperature Conditions

**DOI:** 10.3390/foods9121893

**Published:** 2020-12-18

**Authors:** Theofania N. Tsironi, Nikolaos G. Stoforos, Petros S. Taoukis

**Affiliations:** 1 Laboratory of Food Process Engineering, Department of Food Science and Human Nutrition, Agricultural University of Athens, 11855 Athens, Greece; stoforos@aua.gr; 2 Laboratory of Food Chemistry and Technology, School of Chemical Engineering, National Technical University of Athens, 15780 Athens, Greece; taoukis@chemeng.ntua.gr

**Keywords:** gilthead seabream, European sea bass, yellowfin tuna, kinetic study, predictive models, Arrhenius, cold chain

## Abstract

The objective of this study was the investigation of the effect of variable conditions on quality parameters and the shelf life of fish during frozen storage. Three different fish products were tested, i.e., gilthead sea bream (*Sparus aurata*) fillets, sea bass (*Dicentrarchus labrax*) fillets, and yellowfin tuna (*Thunnus albacares*) slices stored in the range of −5 to −15 °C. The kinetic modeling of different shelf-life indices was conducted. Sensory scoring of frozen fish showed high correlation with color (*L*-value) and total volatile basic nitrogen (TVBN). The temperature dependence of the rates of quality degradation was expressed via the activation energy values, calculated via the Arrhenius equation, and ranged, for the tested quality indices, between 49 and 84 kJ/mol. The estimated kinetic parameters were validated at dynamic conditions and their applicability in real conditions was established, allowing for their practical application as tools for cold chain management.

## 1. Introduction

Freezing is considered one of the most effective fish preservation methods and has been applied increasingly both on shore and on board fishing vessels. Although freezing results in significant extension of shelf life of fish and fish products, sensory, chemical and physical changes of fish quality occur during frozen storage [1]. The quality deterioration of frozen fish depends on extrinsic and intrinsic factors. The determining extrinsic factors are the freezing speed, storage temperature and the temperature fluctuations that often occur in the cold chain, oxygen penetration into the food product, and the mode of thawing or heating of the product. Intrinsic factors are dependent on the biochemical properties of fish and fish products. Enzyme content, the fatty acid profile of the lipid fraction, and the presence of different metabolites, which may be precursors of undesirable components, are responsible for several deteriorative processes [2]. As has long been established, important quality modifications which take place during the frozen preservation of fish are chemical (due to action of naturally occurring enzymes, oxidative and hydrolytic processes in the fats and oils, and denaturation of proteins) and physical (ice crystal formation and desiccation or drying out of the flesh) [3].

When dealing with storage of frozen food, considerable emphasis has been placed on keeping a constant temperature [4]. Temperature observations from recent surveys indicated that, despite good practices, monitoring and control efforts, significant temperature fluctuations occur during distribution, retail and domestic storage of frozen food products. Based on the profile temperatures of frozen fish products, it has been reported that 40% of the total time are over the recommended temperature of −18 °C, varying between −16 and −12 °C, and cases of temperatures above −8 °C are not rare occurrences in retail or domestic storage [5]. The quality degradation of frozen food products, albeit relatively slow, are dependent on storage temperature. It becomes evident that temperature monitoring and control within the cold chain is a prerequisite for effective quality management and optimization. In current practice, this is usually handled by reporting on the packages of frozen food arbitrary disclaimers, stating that if the food is stored always at −18 °C (which in practice rarely occurs), then the expiry date is valid. However, at any other temperature, the food has a significantly shorter shelf life (for example, 2 months for frozen shrimp stored at −8 °C compared to 25 months if stored at −18 °C) [6].

Since storage temperatures in the real cold chain vary, it is necessary to be able to estimate reliably the effect of a known time-temperature scenario that may occur in the real frozen food supply chain on the shelf life of the tested frozen food [7,8]. Despite the many years of research, only a few methods have been employed for determining of quality attributes of frozen fish [2,9,10,11,12,13,14,15,16,17]. The systematic monitoring and modeling of the temperature dependence would be an essential prerequisite for shelf-life optimization and effective management of the cold chain [4,18,19], using conventional temperature control and monitoring and intelligent packaging applications such as TTIs (Time Temperature Integrators) [20,21,22]. Based on reliable kinetic and shelf-life models, the effect of temperature can be monitored and quantitatively translated to food quality, from production to the point of consumption. Such an experimental and systematic modeling approach, essential for effective cold chain management, is lacking in the research literature for important commercial fish.

Gilthead sea bream (*Sparus aurata*) and European sea bass (*Dicentrarhus labrax*) are the two most important cultured fish species in the Mediterranean area. Yellowfin tuna (*Thunnus albacares*) is large pelagic fish, commercially important in the international market. The aim of the study was to investigate and develop mathematical models that describe the effect of variable storage conditions on shelf life and quality indices of frozen gilthead sea bream and sea bass fillets and yellowfin tuna slices, and to comprehensively approach the issue of applicability of the developed models as effective predictive tools for frozen chain monitoring and management.

## 2. Materials and Methods

### 2.1. Raw Material

Marine cultured gilthead sea bream (*Sparus aurata*) and sea bass (*Dicentrarchus labrax*) fillets (90 ± 10 g) were provided by Nireus Aquaculture S.A. (Attica, Greece) in polystyrene boxes, maintained at 0 °C with appropriate quantities of flaked ice, while transferred to the Laboratory of Food Chemistry and Technology (NTUA) within 2–3 h after filleting. Upon receipt, fillets were individually vacuum packed (Boss NT42N, Bad Homburg, Germany) and frozen at −30 °C (Whirlpool AFG 610 M-B chest freezer with convection cooling, Italy). Yellowfin tuna (*Thunnus albacares*) slices (weight: 100 ± 10 g) were individually packed under vacuum and air shipped to the Laboratory of Food Chemistry and Technology of NTUA in polystyrene boxes, with an adequate quantity of dry ice, directly after slicing and freezing at the fish processing location (CEFRICO, Vigo, Spain).

Vacuum packed, frozen fish samples were distributed and stored in controlled temperature cabinets (Sanyo MIR 553, Sanyo Electric Co, Ora-Gun, Gunma, Japan) at isothermal conditions (−5, −8, −12 and −15 °C). Measurements of selected quality parameters were carried out as a function of time during a 14-month period, the obtained data were analyzed and adequate mathematical models were developed. The selected quality indices, sampling frequency and duration of experiments were based on literature review and preliminary experiments conducted on relevant frozen seafood products. The frequency and duration of samplings was selected so as to obtain 5–10 measurements (triplicate samples) during storage for each isothermal experiment [6].

In order to validate the applicability of the mathematical models obtained from the isothermal experiments to the actual time-temperature conditions, a variable scenario (Var) was applied, which consisted of repeated cycles of three successive temperature steps of −12 °C for 24 h, −5 °C for 36 h and −8 °C for 24 h, in a temperature programmable control cabinet (Sanyo MIR 153, Sanyo Electric Co, Ova-Gun, Gunma, Japan). The time-temperature scenario was selected in order to simulate abuse cold chain conditions of frozen storage [5,6]. Temperature in the incubators was constantly monitored with electronic, programmable miniature data loggers (COX TRACER^®^, Belmont, NC, USA).

Measurements were carried out in appropriate time intervals which allow for efficient kinetic analysis of the quality degradation of the tested samples. Before analysis, samples were thawed at room conditions (temperature ranging between 20 and 23 °C) for 60 min, prior to the removal of the external packaging of the samples. Samples were analyzed immediately after thawing.

In order to validate the models for shelf-life prediction during distribution in the real path of frozen fish, a realistic distribution scenario in the current chill chain was simulated. It included an initial stage of 25 days of storage in the packing plant, followed by transportation and storage in a distribution center for 25 days. Subsequently, fish was kept at retail freezers for 50 days, before being purchased by the final consumers that stored them in their domestic freezer for 50 days before consumption [5]. The extent of quality deterioration at the end of each distribution phase was estimated using the validated shelf-life predictive models.

### 2.2. Color Measurement

Color parameters were measured based on CIELab values (*L*-value: lightness, *a*-value: redness and greenness, *b*-value: yellowness and blueness), using the CR-Minolta Chromameter^®^ (Minolta Co., Chuo-Ku, Osaka, Japan) with a measuring diameter of 8 mm. The instrument was standardized under the “C” illuminant condition according to the CIE (Commission International de l’ Eclairage) using a standard white reference tile (calibration plate CR-200, *L* = 97.50, *a* = −0.31, *b* = −3.83). At predetermined times of storage, according to the design, measurements were conducted for fish flesh at two different points [19]. Color was measured on the internal part of fillets (flesh) for gilthead seabream and sea bass. Each measurement was carried out on three independent specimens and the average values were calculated.

### 2.3. Total Volatile Basic Nitrogen (TVBN) Determination

Fish freshness has been evaluated by the measurement of total volatile bases, expressed as Total Volatile Basic Nitrogen (TVBN). Analysis of fish flesh was conducted on a single trichloroacetic acid extraction, followed by nitrogen distillation in a Kjeldahl rapid distillation unit (Büchi 321 Distillation unit, Flawwil, Switzerland) and titration with sulfuric acid [23]. Each measurement was carried out on two independent specimens and the average values were calculated.

### 2.4. Sensory Analysis

The sensory attributes of thawed fish were evaluated in fish samples by a sensory panel of 8 trained evaluators. Fish fillets, individually wrapped in aluminum foil, were broiled at 180 °C for 30 min in a preheated oven. The taste of broiled fish was evaluated and sensory scores were recorded in appropriate forms, reflecting the organoleptic evolution of quality deterioration. An acceptance test was also organized. A rating was assigned separately for each parameter on a 1–9 scale (9 being the highest quality score and 1 the lowest). A sensory score of 5 was taken as the average score of minimum acceptability [24].

### 2.5. Microbiological Analysis

For the enumeration of microbial load (Total Viable Count, TVC) in fish samples during frozen storage, a representative sample (25 g) was transferred to a sterile stomacher bag with 225 mL sterilized Ringer (Merck Ringer Tablets in distilled water) and was homogenized for 60 s with a Stomacher (BagMixer^®^ interscience, Saint-Nom-la-Bretèch, France). A total of 0.1 mL of 10-fold serial dilutions of fish homogenates was spread on the surface of the appropriate media (Plate Count Agar-PCA, Merck, Darmstadt, Germany) in Petri dishes for the enumeration of TVC and incubated at 25 °C for 72 h. Microbial load was expressed as logCFU/g of fish. Each measurement was carried out on two independent specimens and the average values were calculated.

### 2.6. Data Analysis

In order to evaluate the quality changes of frozen fish during storage, the “apparent kinetics” methodological approach was used. This methodology included two main successive calculations [25,26]:

(a) A primary kinetic model, where values obtained from the different measured quality parameters were plotted vs. time for all the tested storage temperatures. The apparent order of quality loss was estimated, based on the least square statistical fit.
(b) A secondary model, which reflects the effect of storage temperature on the parameters of the primary model. The temperature dependence of the deterioration rate constants, *k*, was modeled by the Arrhenius Equation (1)

(1) $$\ln k=\ln k_{ref}-\left(\frac{E_{a}}{R}\right)\left[\frac{1}{T}-\frac{1}{T_{ref}}\right]$$

where *k_ref_* (in d^−1^) is the rate constant of the degradation of the respective quality index at a reference temperature, *T_ref_* (e.g., −18 °C for frozen foods), *T* is the temperature (in K), *E_a_* is the activation energy of the studied action (in J/mol) and *R* is the universal gas constant. The *E_a_* values were estimated from the slope of Arrhenius plots of ln*k* vs. (1/*T_ref_* − 1/*T*), by linear regression [27,28].

For mathematical model validation at variable conditions, two alternative methodologies were compared for the estimation of the predicted quality indices (i.e., *L*-value, TVBN value and sensory scoring), at predetermined points of the variable time-temperature scenario. The quality level of fish was determined using the Arrhenius-based kinetics developed from the data obtained from the isothermal experiments. To demonstrate the integrated effect of temperature variability on product quality, the term of effective temperature *T_eff_* was introduced for the first method (Method #1) [29]. *T_eff_*, which is defined as the constant temperature that results in the same quality value as the variable temperature distribution over the same time period, is based on the Arrhenius model and integrates in a single value the effect of the variable temperature profile at a specific time of the time-temperature scenario. *T_eff_* is calculated by the Arrhenius model (Equation (1)) for *k* = *k_eff_* (i.e., the value of the rate of the quality loss reaction at the effective temperature *T_eff_*), as estimated by Equation (2) [29].

(2) $$k_{ref}\cdot \underset{i}{\sum }\left[exp\left(-\frac{E_{a}}{R}\cdot \left(\frac{1}{T_{i}}-\frac{1}{T_{ref}}\right)\right)\cdot t_{i}\right]=k_{eff}\cdot t_{tot}$$

For the second method (Method #2), the *T*(*t*) function was discretized in small time increments *t_i_* of constant temperature *T_i_*. Data analysis was based on the determination of the quality status of fish at any *t_i_*, by calculating the quality deterioration using the developed models for each Δ*t* (using the actual time-temperature conditions at each *t_i_*). In this case, for the determination of the loss of a quality factor (color change, TVBN or sensory scoring, *s*), the following equations were used (Equations (3)–(5)),

(3) $$L_{i+1}=L_{i}-k_{L}\cdot \Delta t$$

(4) $$C_{TVBN,i+1}=C_{TVBN,i}\cdot e^{k_{TVBN}\cdot \Delta t}$$

(5) $$s_{i+1}=s_{i}-k_{s}\cdot \Delta t$$

where *k* is the rate constant for color change (*L*-value decrease), TVBN increase or sensory scoring decrease, *i* and *i* + 1 refer to time, as *t_i_*_+1_ = *t_i_* + Δ*t*, *C_TVBN_* is the TVBN concentration in fish flesh, *s* is the sensory scoring and *L* is the *L*-value at the respective time indicated by the subscript. The rate *k* is a function of storage temperature *k*(*t*) and is calculated by Equation (1) for *T_i_* [30,31].

The rates of quality deterioration (TVBN, sensory evaluation) that were calculated using the proposed mathematical methods along with the kinetics derived from the isothermal experiments were compared to the quality level of fish fillets that were experimentally measured at predetermined points of frozen storage at the Var scenario.

The comparison between the experimental (actual) and predicted (calculated by the mathematical food kinetic models) quality indices was based on the accuracy (*A_f_*) and bias (*B_f_*) factors (Equations (6) and (7))

(6) $$A_{f}=10^{\frac{\sum \left|\log(y_{\mathrm{predicted}}/y_{\mathrm{experimental}})\right|}{n}}$$

(7) $$B_{f}=10^{\frac{\sum \log(y_{\mathrm{predicted}}/y_{\mathrm{experimental}})}{n}}$$

where *n* is the number of observations, and the relative error (RE) calculated by Equation (8) for each one of the obtained *y_i_* values. Perfect agreement between the predicted and the respective observed values is represented with *A_f_* and *B_f_* values of 1 [22,32].

(8) $$\mathrm{RE}=\frac{\left(y_{\mathrm{observed}}-y_{\mathrm{predicted}}\right)}{y_{\mathrm{predicted}}}$$

## 3. Results

### 3.1. Effect of Frozen Storage on Appearance and Color of Fish Fillets

**Figure 1:**
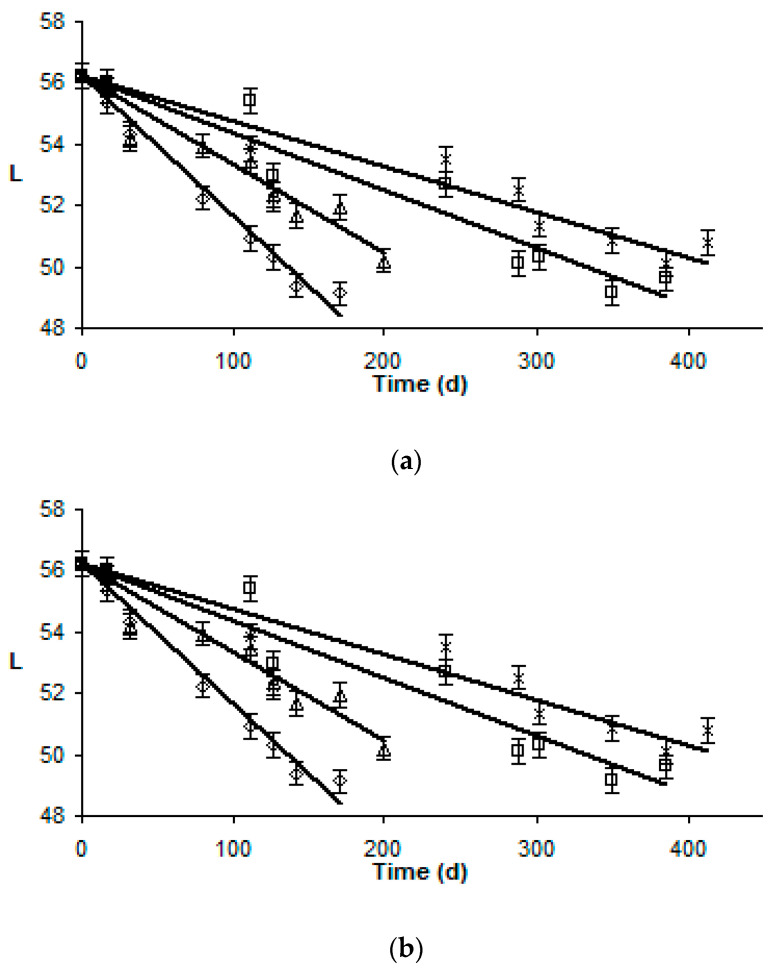

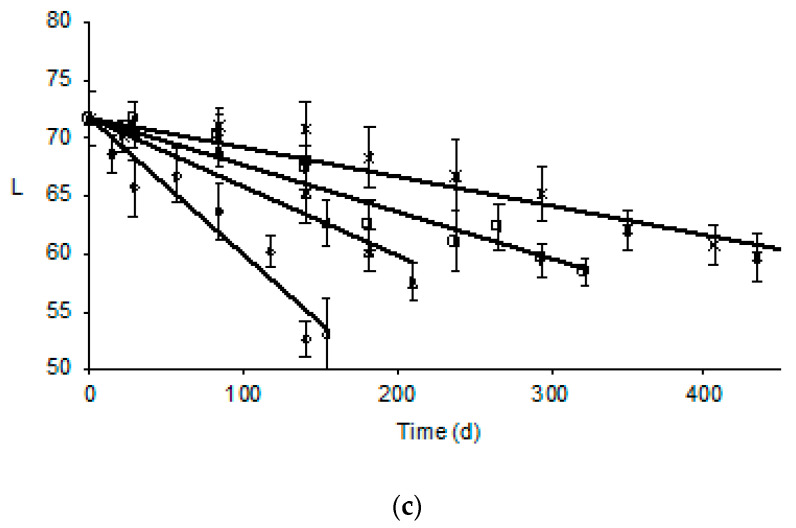
Changes of color (*L*-value) of thawed (**a**) gilthead sea bream fillets, (**b**) sea bass fillets and (**c**) yellowfin tuna slices during storage at ◊ −5, ∆ −8, ☐ −12 and ∗ −15 °C. (Error bars indicate standard error of measurements of three different samples.) (1**a**: *R*^2^_−5°C_ = 0.822, *R*^2^_−8°C_ = 0.820, *R*^2^_−12°C_ = 0.939, *R*^2^_−15°C_ = 0.937; 1**b**: *R*^2^_−5°C_ = 0.986, *R*^2^_−8°C_ = 0.938, *R*^2^_−12°C_ = 0.852, *R*^2^_−15°C_ = 0.932; 1**c**: *R*^2^_−5°C_ = 0.822, *R*^2^_−8°C_ = 0.820, *R*^2^_−12°C_ = 0.939, *R*^2^_−15°C_ = 0.937).

At zero storage time, gilthead sea bream and sea bass had white and shiny flesh. Lightness values were initially 66.7 ± 2.2, 56.3 ± 1.4 and 71.7 ± 2.4 for gilthead sea bream, sea bass and yellowfin tuna, respectively, as indicated in Figure 1.

The *L* value of thawed fish flesh showed a significant decrease at the highest storage temperatures, as shown at Figure 1, and was a good quality index, as darkened fish flesh was associated with poor quality. The decrease in the L value of fish flesh during storage was adequately modeled by an apparent zero order reaction (Equation (3)).

Temperature dependence of the rates of color degradation was adequately described by Arrhenius kinetics in the temperature range studied. Changes of *L*-value showed *E_a_* values and 95% confidence range 48.9 ± 2.1 kJ/mol (*R*^2^ = 0.997), 64.4±6.4 kJ/mol (*R*^2^ = 0.980) and 83.9±10.4 kJ/mol (*R*^2^ = 0.970) for gilthead sea bream fillets, sea bass fillets and yellowfin tuna slices, respectively (Table 1). Other color parameters evaluated (i.e., *a*-value and *b*-value) showed slight changes during storage, within the calculated sample variability, during the isothermal temperature conditions or not dependent changes on storage temperature. Thus, other color changes were not modelable indices of frozen fish quality deterioration.

**Figure 2:**
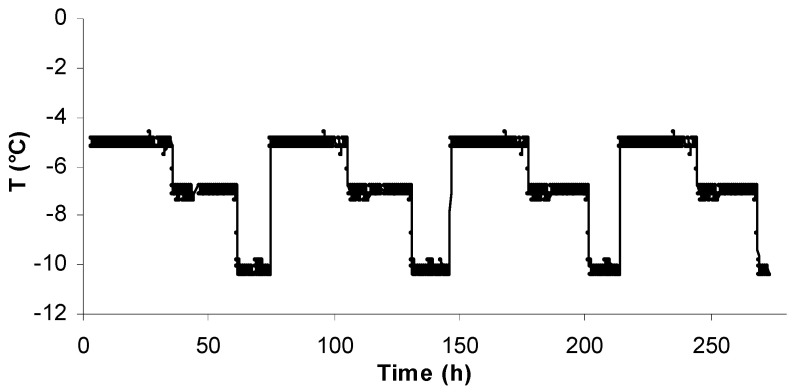
Temperature profile of the non-isothermal experiment.

**Figure 3:**
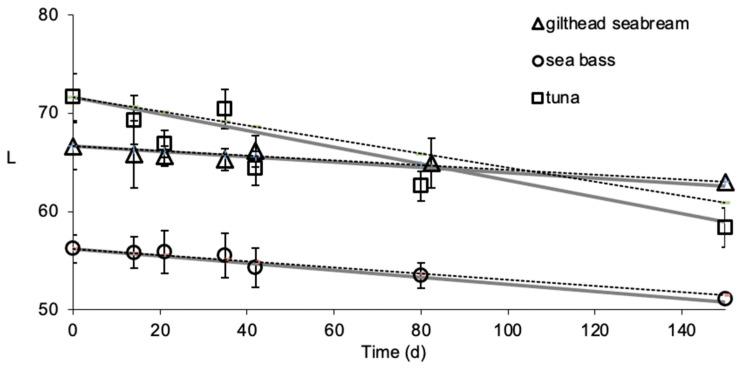
Comparison of experimental and predicted changes in *L*‑value of thawed gilthead sea bream fillets, sea bass fillets and yellowfin tuna slices at non-isothermal conditions (Figure 2). The gray solid lines indicate predictions by Method #1, the black dashed lines indicate the predictions by Method #2, and the data points present the experimental values. (Error bars indicate standard error of measurements of three different samples.)

The *L*-value was determined during non-isothermal storage (Figure 2) and was compared with the predicted values (Method #1 and Method #2), validating the applicability of the mathematical models at non-isothermal temperature conditions (Figure 3). *T_eff_* was calculated as equal to −7.9 °C for gilthead seabream fillets, −7.8 °C for sea bass fillets and −7.8 °C for yellowfin tuna slices (well within the temperature range studied). For the calculations based on Method #1, the RE,*_L_*_-value_ ranged from −0.008 to 0.010 for gilthead seabream fillets, −0.011 to 0.008 for sea bass fillets and −0.065 to 0.019 for yellowfin tuna slices. For the calculations based on Method #2, RE,*_L_*_-value_ ranged from 0.007 to 0.008 for gilthead sea bream fillets, −0.011 to 0.008 for sea bass fillets and −0.066 to 0.019 for yellowfin tuna slices. The calculated relative error revealed that all points fall within the acceptable prediction zone as defined by [32], i.e., −0.3 < RE < 0.15. The *A_f_* and *B_f_* values indicated that there was a satisfactory agreement between predicted and observed *L*-values (Table 2 and Table 3). The *B_f_* values were within the boundaries of 0.7 (fail-safe) to 1.15 (fail-dangerous) [33].

### 3.2. Effect of Frozen Storage on TVBN

**Figure 4:**
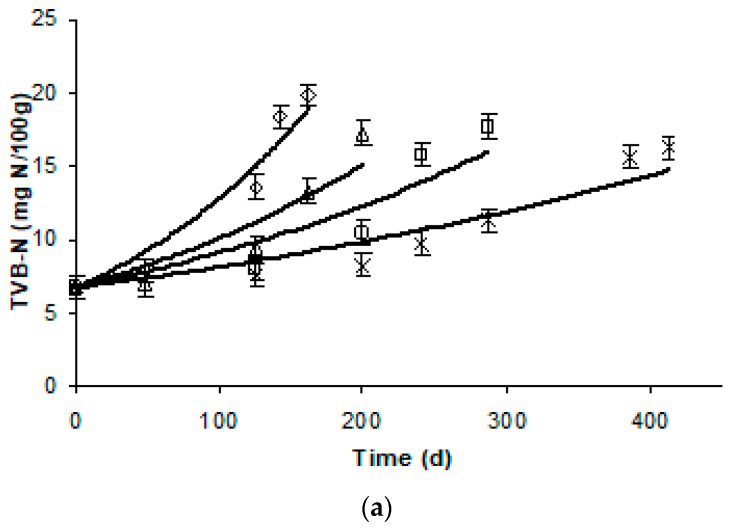

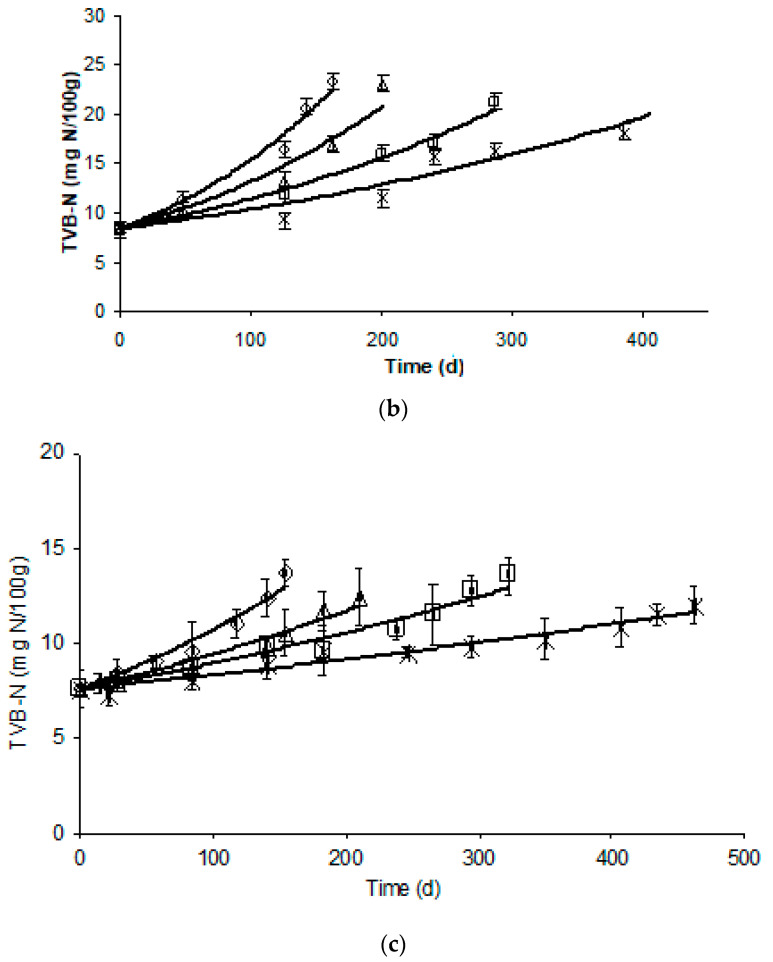
Changes in the total volatile basic nitrogen (TVBN) of thawed (**a**) gilthead sea bream fillets, (**b**) sea bass fillets and (**c**) yellowfin tuna slices during storage at ◊ −5, ∆ −8, ☐ −12 and ∗ −15 °C. (Error bars indicate standard error of measurements of two different samples.) (1**a**: *R*^2^_−5°C_ = 0.963, *R*^2^_−8°C_ = 0.924, *R*^2^_−12°C_ = 0.885, *R*^2^_−15°C_ = 0.931; 1**b**: *R*^2^_−5°C_ = 0.983, *R*^2^_−8°C_ = 0.963, *R*^2^_−12°C_ = 0.990, *R*^2^_−15°C_ = 0.930; 1**c**: *R*^2^_−5°C_ = 0.964, *R*^2^_−8°C_ = 0.923, *R*^2^_−12°C_ = 0.885, *R*^2^_−15°C_ = 0.931.)

Changes in TVBN values are shown in Figure 4a–c. TVBN was initially 6.8 and 8.4 mg N/100 g for gilthead sea bream and sea bass fillets and increased with storage time up to approximately 20 and 23 mg N/100 g, respectively. For yellowfin tuna slices, initial TVBN was 7.6 mg N/100 g and increased with storage time up to approximately 18 mg N/100 g. TVBN values were modeled with apparent first-order equations (Equation (4), *R*^2^ > 0.90 for all experiments).

The temperature dependence of the TVBN formation rates in fish samples was adequately described by Arrhenius kinetics in the temperature range studied, showing activation energy, *E_a_*, values and a 95% confidence range of 65.9 ± 7.0 (*R*^2^ = 0.978), 60.8 ± 2.7 (*R*^2^ = 0.996) and 69.9 ± 8.5 kJ/mol (*R*^2^ = 0.971) for gilthead seabream fillets, sea bass fillets and yellowfin tuna slices, respectively. These values were close to the respective *E_a_* for color change, indicating similar temperature dependence to the chemical quality parameters (Table 1).

**Figure 5:**
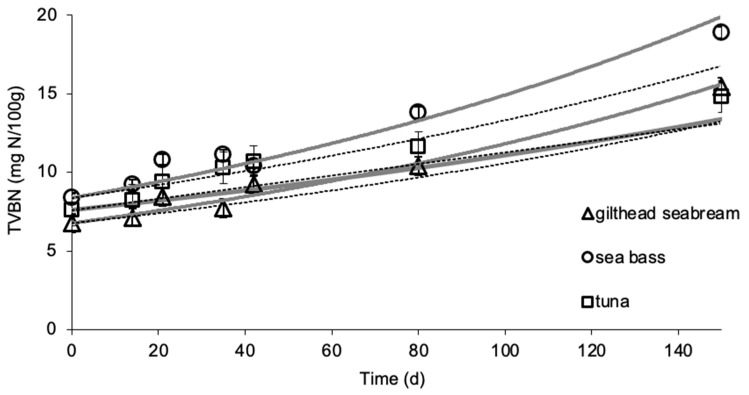
Comparison of experimental and predicted changes in the TVBN of gilthead sea bream fillets, sea bass fillets and yellowfin tuna slices at the temperature profile of the non-isothermal experiment. The gray solid lines indicate predictions by Method #1, the black dashed lines indicate predictions by Method #2, and the data points present the experimental values. (Error bars indicate standard error of measurements of two different samples.)

TVBN changes were also measured during storage experiment at dynamic conditions. The results, as shown at Figure 5, indicated a satisfactory agreement between the experimental data and the prediction based on the developed kinetic models, validating their applicability at variable conditions. For Method #1, the RE,_TVBN_ values ranged from −0.030 to 0.140 for gilthead seabream fillets, 0.022 to 0.046 for sea bass fillets and 0.023 to 0.144 for yellowfin tuna slices. For Method #2, the RE,_TVBN_ ranged from −0.027 to 0.144 for gilthead sea bream fillets, 0.022 to 0.145 for sea bass fillets and −0.104 to −0.032 for yellowfin tuna slices. The calculated relative errors revealed that all points fall within the acceptable prediction zone as defined by [32], i.e., −0.3 < RE < 0.15. The *A_f_* and *B_f_* values indicated that there was a satisfactory agreement between predicted and observed *L*-values (Table 2 and Table 3). The *B_f_* values were within the boundaries of 0.7 (fail-safe) to 1.15 (fail-dangerous) [33].

### 3.3. Sensory Evaluation

Sensory scorings were evaluated by a sensory panel and the shelf life of frozen fish samples was determined. Sensory scoring declined with storage time and temperature. The main parameter that, according to the panelists, affected the overall acceptability of the fish samples was the taste of the broiled fish. Fish flesh became darker and yellowish during storage, especially for samples stored at −5 and −8 °C. Fish fillets or slices stored isothermally at −12 and −15 °C had acceptable appearance, as indicated by the sensory panel, for approximately 9 months and 1 year, respectively. Sensory scorings were modeled by apparent zero order reactions (Equation (5)) and they were good quality indexes for the tested frozen fish products, as the calculated *E_a_* values were in agreement with the ones for flesh color (*L*-value) and chemical quality parameters (TVBN), i.e., *E_a_* values of 65.2 ± 2.8 kJ/mol (*R*^2^ = 0.996) and 64.5 ± 2.7 kJ/mol (*R*^2^ = 0.997) for taste and 65.3 ± 14.9 kJ/mol (*R*^2^ = 0.999) and 64.3 ± 0.5 kJ/mol (*R*^2^ = 0.998) for overall acceptability for gilthead sea bream and sea bass fillets, respectively. For yellowfin tuna slices, the respective *E_a_* values were calculated as 69.4 ± 3.5 (*R*^2^ = 0.995) and 77.9 ± 13.7 kJ/mol (*R*^2^ = 0.942) for taste and overall acceptability (Table 1). It has been reported that the ± 20 kJ/mol of the activation energy of the selected quality index of the target food product could be considered as similar temperature dependence [6].

**Figure 6:**
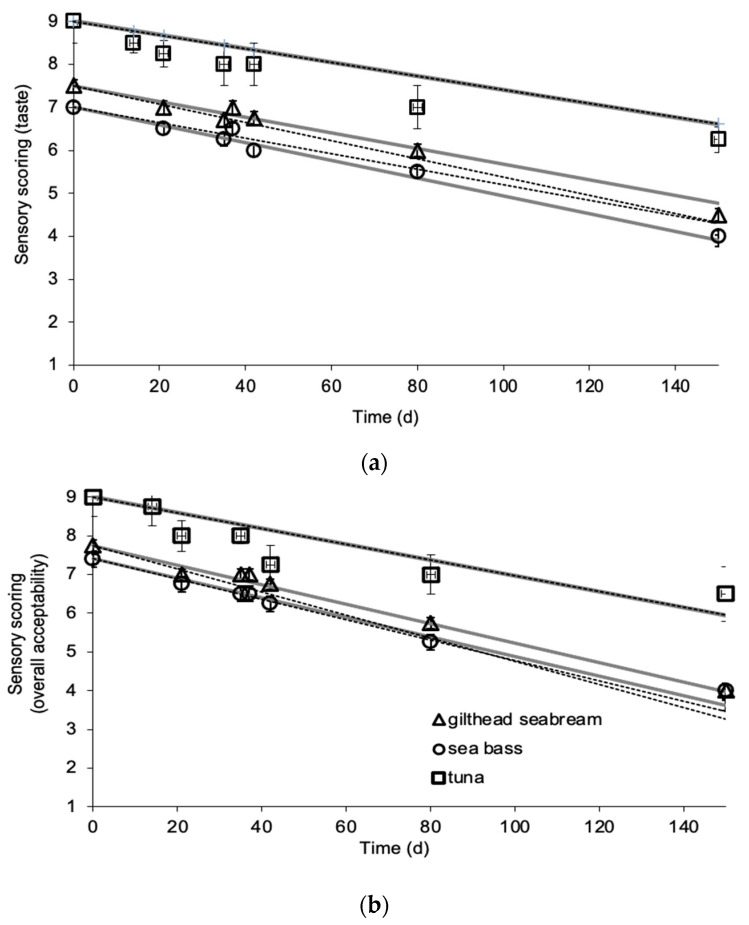
Comparison of experimental and predicted changes in (**a**) taste and (**b**) overall acceptability of gilthead sea bream fillets, sea bass fillets and yellowfin tuna slices at the temperature profile of the non-isothermal experiment. The gray solid lines indicate predictions by Method #1, the black dashed lines indicate the predictions by Method #2, and the data points present the experimental values. (Error bars indicate standard error of scoring of eight panelists.)

The results of the sensory evaluation, as indicated by taste and overall acceptability scores, during the non-isothermal experiments, as presented in Figure 6a,b, are in agreement with the ones calculated by the models developed by the isothermal experiments, validating the applicability of the established kinetic models in the dynamic temperature condition of the chill chain. For Method #1, the RE,_taste_ values ranged from 0.036 to 0.075 for gilthead seabream fillets, −0.073 to −0.010 for sea bass fillets and −0.104 to −0.033 for yellowfin tuna slices. The respective RE,_ov_acceptability_ values ranged from 0.044 to 0.109 for gilthead seabream fillets, −0.029 to 0.077 for sea bass fillets and −0.124 to 0.084 for yellowfin tuna slices. For Method #2, the RE,_taste_ value ranged from −0.025 to 0.062 for gilthead sea bream fillets, −0.073 to −0.010 for sea bass fillets and −0.104 to −0.032 for yellowfin tuna slices. The respective RE,_ov_acceptability_ values ranged from 0.044 to 0.110 for gilthead sea bream fillets, −0.028 to 0.076 for sea bass fillets and −0.124 to 0.084 for yellowfin tuna slices. The calculated relative errors revealed that all points fall within the acceptable prediction zone as defined by [32], i.e., −0.3 < RE < 0.15. The *A_f_* and *B_f_* values indicated that there was a satisfactory agreement between predicted and observed *L*-values (Table 2 and Table 3). The *B_f_* values were within the boundaries of 0.7 (fail-safe) to 1.15 (fail-dangerous) [33].

The shelf life of frozen gilthead sea bream and sea bass fillets and yellowfin tuna slices, determined based on sensory scoring (limit = score 5 for overall acceptability) and on TVBN value (limit = 20 mg N/100 g), is shown in Table 4. The estimated shelf-life values are in agreement with previous studies on frozen albacore tuna, where TVBN correlated satisfactorily with the sensory rejection [13] and vacuum packed, frozen, and scaled whitefish [10]. In the latter study, vacuum packaging inhibited lipid oxidations reactions and extended frozen fish shelf life at −12 and −25 °C. TVBN has also been reported as an adequate quality index for frozen cod by a relevant study [12]. Dimethylamine and formaldehyde content correlated satisfactorily with a texture sensory score of frozen red hake; these objective tests were reported as useful for predicting textural quality and thus shelf life [9].

Similar Arrhenius type models have been developed for blueshark slices and arrow squid, with the aim of providing a TTI-based system for the effective management of the frozen seafood supply chain [8]. These models were validated in the real cold chain through a pilot field study [22]. The *E_a_* values of the quality deterioration rates of frozen gilthead sea bream fillets, sea bass fillets and yellowfin tuna slices were lower than the respective values reported for frozen shrimp [19], indicating a higher temperature sensitivity of frozen shrimp compared to the fish products studied in the present article. The *E_a_* ranged between 118 and 156 kJ/mol for the tested quality indices for frozen, whole, and unpeeled shrimp. The higher values, compared to the respective *E_a_* in the present study, may be attributed to the different mechanisms of quality deterioration in the case of shrimp, i.e., mainly enzymatic browning resulting in blackening, which significantly limits the shelf life at higher storage temperatures (−5 °C). A similar approach, based on Arrhenius kinetics, has been previously reported for the shelf-life modelling of frozen cod in the temperature range of −12 to −30 °C [4]. A prediction model of shelf life by fractal dimension has been developed to observe the porous microstructure that results from the ice crystal formation in frozen hairtail meat and tilapia [34,35]. The Gompertz and polynomial models have been used for the evaluation of the correlations between TVBN and trimethylamine (TMA) formation in mackerel during frozen storage [36].

### 3.4. Effect of Frozen Storage on Microbial Load

**Figure 7:**
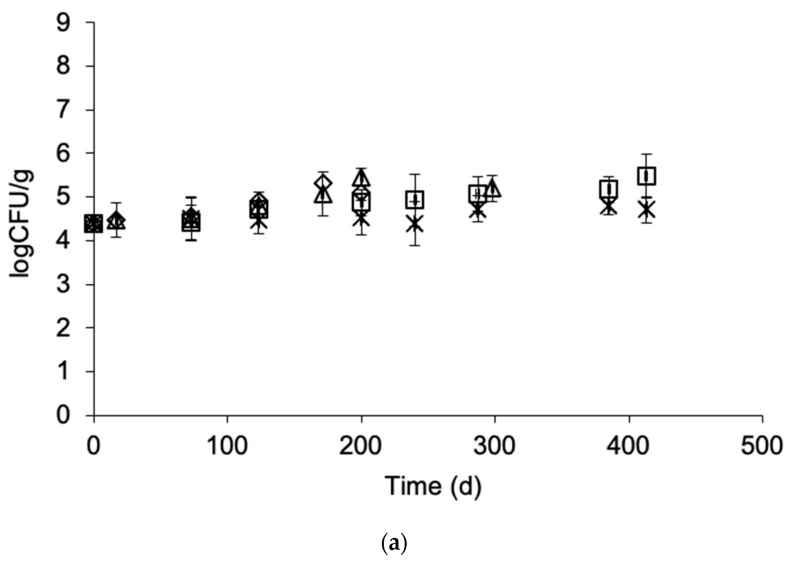

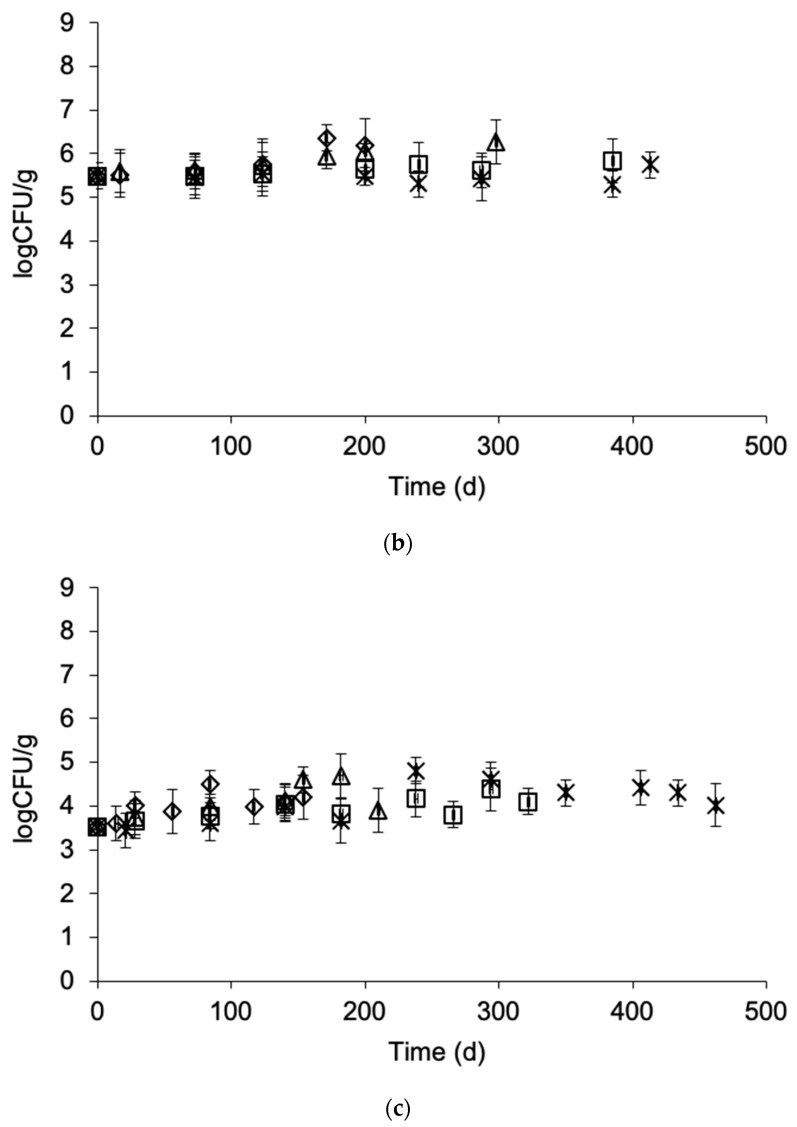
Development of total viable count in (**a**) gilthead sea bream fillets, (**b**) sea bass fillets and (**c**) yellowfin tuna slices during storage at ◊ −5, ∆ −8, ☐ −12 and ∗ −15 °C. (Error bars indicate standard error of measurements of two different samples.)

Total viable count in frozen gilthead sea bream fillets, European sea bass fillets and yellowfin tuna slices was 4.4 ± 0.2, 5.5 ± 0.3 and 3.5 ± 0.2 logCFU/g, respectively. These values are in accordance with the respective microbial count levels reported in the literature for frozen fish [33,37]. No significant growth was observed during the storage of frozen fish samples at any of tested temperatures. The final total viable counts ranged from 4.4 to 5.5, 5.8 to 6.3 and 3.9 to 4.2 logCFU/g for gilthead sea bream fillets, European sea bass fillets and yellowfin tuna slices, respectively (Figure 7a–c). At the end of the storage period, no “spoilage” characteristics were reported by the sensory panel.

### 3.5. Application of the Validated Models for Shelf-Life Prediction during the Distribution of Frozen Fish

**Figure 8:**
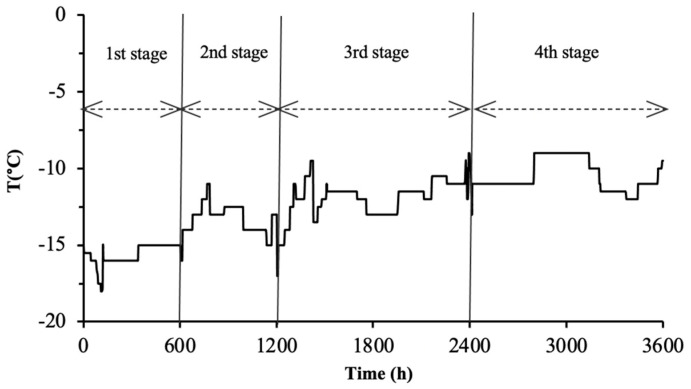
Indicative temperature profile of the distribution of frozen fish in the real chill chain. The total distribution time was 150 days. (1st stage: packing plant storage, 2nd stage: transportation-distribution center, 3rd stage: retail storage, 4th stage: domestic storage).

The objective of the present study was to develop and validate reliable kinetic models of selected quality indices in order to allow for the quality and shelf-life estimations of different fish products during non-isothermal distribution and storage, in the actual frozen distribution path. The level of quality deterioration at the end of each distribution and storage phase of the tested realistic distribution scenario (Figure 8) was estimated using the validated shelf-life predictive models, and the *T_eff_* calculation approach (Method #1), as presented in Table 5. Although Method #2 is mathematically more rigorous compared to Method #1, the latter was selected for further use due to its simplicity. Method #1 is not dynamic in nature; however, in the limited temperature range that occurred during frozen storage, it gives acceptable results.

At the end of the simulated cycle, i.e., assumed as the time of consumption, the estimated remaining shelf life (RSL) for gilthead sea bream fillets, sea bass fillets and yellowfin tuna slices (at −15 °C) according to sensory scoring was 204, 197 and 217 days, respectively. The nominal RSL based on the “use by” date, which does not take into account the food product time-temperature history, would be higher than 250 days (i.e., 265, 258 and 295 days for gilthead sea bream fillets, sea bass fillets and yellowfin tuna slices, respectively). Under this context, the potential of using validated shelf-life predictive models as reliable tools for the quality assessment of products at the given point of the frozen distribution chain is substantiated. These models in combination with adequate TTI smart labels could allow for better management and optimization of the cold chain, from manufacture to the point of consumption.

## 4. Conclusions

The process of freezing as a method of preserving fish quality has been long established. However, temperature fluctuations may occur throughout the production and distribution chain. Overall, in this study, a systematic modeling of quality deterioration of frozen fish at variable temperature conditions was achieved.

The protocols for the shelf-life testing of foods consist of defining the quality parameters which deteriorate faster with time and the mathematical modeling of these indices. In several cases, shelf-life testing experiments are accelerated in order to evaluate the effect of different formulation and processing parameters on the quality and shelf life of the tested food product. Accelerated shelf-life testing conducted at elevated isothermal temperatures for frozen products has been used extensively for several decades by industry and government agencies [38]. In addition, temperature fluctuations may occur in distribution and retail holding for frozen storage. Thus, kinetic studies at several temperatures within that range are necessary to predict its shelf life. The applicability of the developed models was evaluated via non-isothermal experiments using independent product batches, in order to validate the reliability of the shelf-life models for the frozen fish production sector at the temperature range of −15 to −5 °C, which lies within the reported temperature conditions of the actual supply chain of frozen foods [5]. The validated models combined with the application of TTI may be an effective tool for frozen fish quality monitoring during their transportation and storage.

## Figures and Tables

**Table 1:** Activation energy values (*E_a_*) for the quality deterioration rates of frozen gilthead sea bream fillets, sea bass fillets and yellowfin tuna slices stored in the range of −5 to −15 °C (calculated value ± 95% confidence intervals based on the statistical variation of the kinetic parameters of the Arrhenius model—regression analysis).

		*L*-Value	TVBN	Taste	Ov. Acceptability
Gilthead sea bream fillets	*E_a_* (kJ/mol)	48.9 ± 2.1 ^a^	65.9 ± 7.0 ^a^	65.2 ± 2.8 ^a^	65.3 ± 14.9 ^a^
	*R* ^2^	0.997	0.978	0.996	0.999
Sea bass fillets	*E_a_* (kJ/mol)	64.4 ± 6.4 ^a^	60.8 ± 2.7 ^a^	64.5 ± 2.7 ^a^	64.3 ± 0.5 ^a^
	*R* ^2^	0.980	0.996	0.997	0.998
Yellowfin tuna slices	*E_a_* (kJ/mol)	83.9 ± 10.4 ^a^	69.9 ± 8.5 ^a^	69.4 ± 3.5 ^a^	77.9 ± 13.7 ^a^
	*R* ^2^	0.970	0.971	0.995	0.942

^a^ Different superscripts in the same raw indicate significant differences (*p* < 0.05).

**Table 2:** Performance evaluation of the mathematical models for the quality parameters of frozen gilthead sea bream fillets, sea bass fillets and yellowfin tuna slices (Method #1).

		*L*-Value	TVBN	Taste	Ov. Acceptability
Gilthead sea bream fillets	*A_f_*	1.0012	1.0018	1.0061	1.0075
	*B_f_*	1.0015	0.9277	0.9447	0.9212
	RE	−0.008 to 0.010	−0.030 to 0.140	0.036 to 0.075	0.044 to 0.109
Sea bass fillets	*A_f_*	1.0002	1.0051	1.0062	1.0048
	*B_f_*	1.0010	0.9079	1.0329	0.9743
	RE	−0.011 to 0.008	0.022 to 0.046	−0.073 to −0.010	−0.029 to 0.077
Yellowfin tuna slices	*A_f_*	1.0042	1.0046	1.0032	1.0029
	*B_f_*	1.0103	0.9093	0.9725	0.9606
	RE	−0.0065 to 0.019	0.023 to 0.144	−0.104 to −0.033	−0.124 to 0.084

**Table 3:** Performance evaluation of the mathematical models for the quality parameters of frozen gilthead sea bream fillets, sea bass fillets and yellowfin tuna slices (Method #2).

		*L*-Value	TVBN	Taste	Ov. Acceptability
Gilthead sea bream fillets	*A_f_*	1.0012	1.0019	1.0055	1.0076
	*B_f_*	1.0016	0.9292	0.9660	0.9199
	RE	−0.007 to 0.008	−0.027 to 0.144	−0.025 to 0.062	0.044 to 0.110
Sea bass fillets	*A_f_*	1.0003	1.0050	1.0064	1.0045
	*B_f_*	1.0014	0.9082	1.0337	0.9740
	RE	−0.011 to 0.008	0.022 to 0.145	−0.073 to −0.010	−0.028 to 0.076
Yellowfin tuna slices	*A_f_*	1.0030	1.0056	1.0047	1.0052
	*B_f_*	1.0094	0.9086	0.9821	0.9832
	RE	−0.066 to 0.019	−0.104 to −0.032	−0.104 to −0.032	−0.124 to 0.084

**Table 4:** Shelf life (days) of frozen gilthead sea bream fillets, sea bass fillets and yellowfin tuna slices stored at different temperatures.

		−5 °C	−8 °C	−12 °C	−15 °C	−18 °C
Gilthead sea bream fillets	TVBN (limit = 15 mg N/100 g)	130	182	287	408	586
	Sensory (limit = 5 score for overall acceptability)	134	186	293	415	594
Sea bass fillets	TVBN (limit = 20 mg N/100 g)	140	191	291	403	563
	Sensory (limit = 5 score for overall acceptability)	133	185	290	408	580
Yellowfin tuna slices	TVBN (limit = 22 mg N/100 g)	156	212	323	448	624
	Sensory (limit = 5 score for overall acceptability)	152	208	320	445	623

**Table 5:** Quality deterioration and remaining shelf life (RSL according to sensory scoring (limit = 5 for overall acceptability) of frozen gilthead sea bream fillets, sea bass fillets and yellowfin tuna slices at the end of each stage of the realistic time-temperature profile (Figure 7).

	1st Stage Duration: 25 Days	2nd Stage Duration: 25 Days	3rd Stage Duration: 50 Days	4th Stage Duration: 50 Days
*T_eff_* (°C)	−15.6	−13.3	−11.8	−10.4
Frozen gilthead sea bream fillets				
Sensory Scoring	8.77	8.48	7.78	6.96
RSL_predicted_ (d)	391	361	288	204
Frozen sea bass fillets				
Sensory Scoring	8.72	8.25	7.61	6.85
RSL_predicted_ (d)	384	353	281	197
Frozen yellowfin tuna slices				
Sensory Scoring	8.79	8.50	7.80	6.95
RSL_predicted_ (d)	421	389	311	217
